# Supplementary material for: Influence of Monomer Size on CO2 Adsorption and Mechanical Properties in Microporous Cyanate Ester Resins
Source: Polymers (Basel). 2025 Jan 9;17(2):148. doi: 10.3390/polym17020148 (PMC11769292; doi:10.3390/polym17020148)
Supplement: Supplementary file 1 [file polymers-17-00148-s001.zip › polymers-3395605-supplementary.pdf]

Influence of Monomer Size on CO_2 Adsorption and Mechanical Properties in Microporous Cyanate Ester Resins

Yukun Bai [1], Gota Kikugawa [2] and Naoki Kishimoto [1,*]

1 Department of Chemistry, Graduate School of Science, Tohoku University, Aramaki, Aoba-ku, Sendai 980-8578, Japan; bai.yukun.p8@dc.tohoku.ac.jp (Y. B)
2 Institute of Fluid Science, Tohoku University, Katahira, Sendai 980-8577, Japan; kikugawa@tohoku.ac.jp (G. K)

* Correspondence: kishimoto@tohoku.ac.jp

Supporting Information

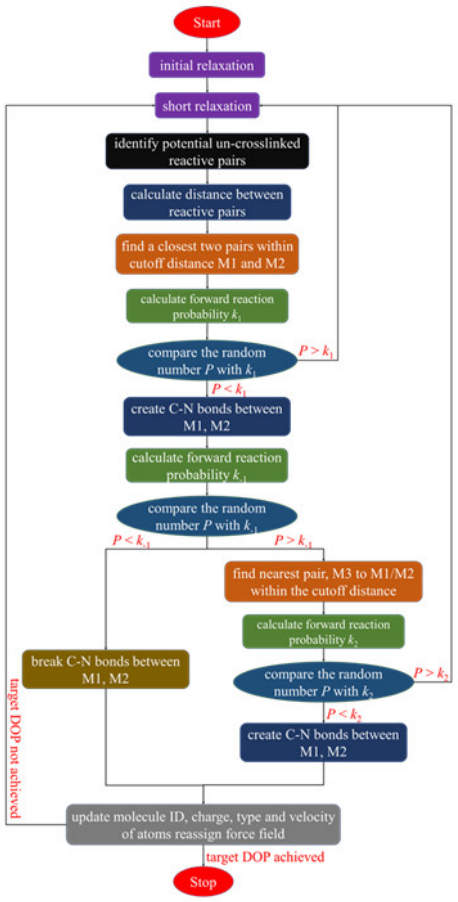

Cutoff distance for reaction: 3.5 Å
P : A random number from 0-1

$$k = Aexp\left(-\frac{E_a}{RT}\right)$$

A is the acceleration factor that accelerates the simulation (in this study, we set A to 10^{15}); E_a is the activation energy for the forward and backward reactions, at here, E_a for forward reaction is 62.46 kcal/mol for k_1, and 52.48 kcal/mol for k_2; As the backward reaction, E_a is 43.76 kcal/mol for k_{-1}. R is the universal gas constant; and T is the local temperature at the reaction sites, which was calculated based on the velocity of the reaction atoms.

Figure S1. The crosslinking flowchart and criteria used in this study reported in our previous study[1].

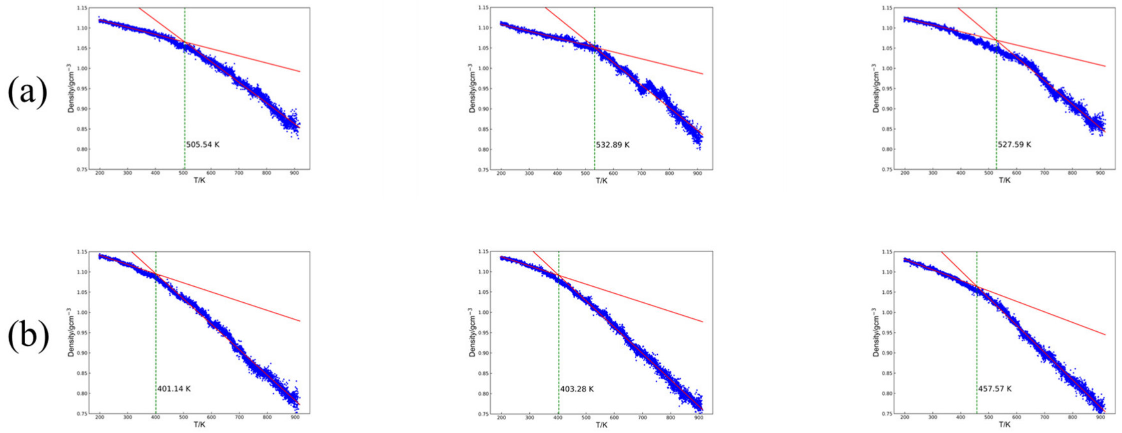

Figure S2. The calculated T_g for all systems in this study (a) S-small; (b) S-large.

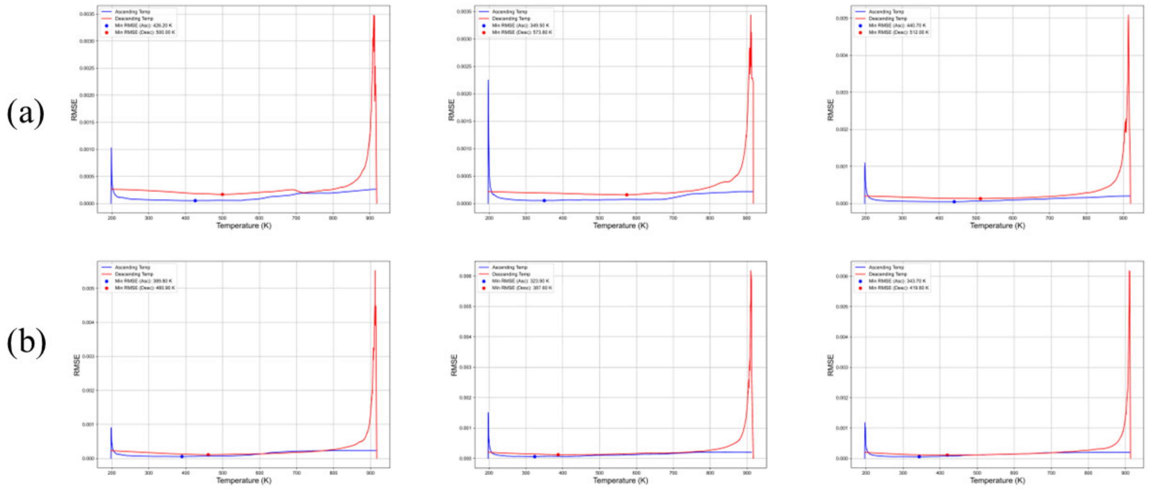

Figure S3. RMSEs-temperature plot for (a) S-small; (b) S-large with lowest point. Color code: blue for low temperature region; red for high temperature region.

Structure information for CO_2 used in this study.

1 C1	0.0000	0.0000	0.0000	C
2 O2	0.0000	0.0000	1.1607	O
3 O3	0.0000	0.0000	-1.1607	O

Structure information for CO2 and triazine ring complex.

C	-0.69450700	5.24213400	-1.25381300
C	-0.91294600	3.86838800	-1.32411700
C	-1.15630900	3.17114300	-0.15092100
C	-1.20055100	3.80112000	1.08310700
C	-0.98109100	5.17563900	1.14238000
C	-0.72636300	5.89624800	-0.02311800
H	-0.49961100	5.80074400	-2.16273600
H	-0.89299000	3.34028800	-2.27011700
H	-1.40476000	3.22003700	1.97481200
H	-1.01072600	5.68205800	2.10093900
H	-0.55585600	6.96607100	0.02687100
O	-1.45179700	1.80057200	-0.21563000
C	-0.45095000	0.91128500	-0.16526900
C	0.12831800	-1.23851500	-0.12298900
C	1.69512100	0.34084200	-0.06534300
N	0.80218500	1.33467300	-0.10894300
N	-0.86297000	-0.35906100	-0.17678200
N	1.43230300	-0.95926500	-0.06924600
O	2.96711600	0.75386300	-0.01444600
O	-0.15660100	-2.54652900	-0.12155900
C	3.99192600	-0.20218500	0.05524000
C	4.38976500	-0.68013000	1.29474600
C	4.63285500	-0.57822300	-1.11496100
C	5.45760300	-1.57165400	1.35946700
H	3.86745900	-0.35890200	2.18826300
C	5.70064400	-1.47011600	-1.03977400
H	4.29657500	-0.17674100	-2.06373100
C	6.11257900	-1.96880700	0.19462000
H	5.77726700	-1.95560900	2.32207700
H	6.20973000	-1.77416900	-1.94785700
H	6.94382200	-2.66314600	0.24947100
C	-1.50287700	-2.94325300	-0.12757400
C	-2.08992700	-3.29445000	-1.33338100
C	-2.18570600	-3.04123400	1.07565900
C	-3.40649700	-3.74886600	-1.33267200
H	-1.52306400	-3.20393800	-2.25243200
C	-3.50261800	-3.49547500	1.06547200
H	-1.69614400	-2.75440100	1.99859600
C	-4.11449800	-3.84746900	-0.13658100
H	-3.88065500	-4.01770700	-2.27002000
H	-4.04946400	-3.56944100	1.99905000
H	-5.14130600	-4.19602500	-0.14105000
C	-3.70992300	-0.11515400	0.49704500
O	-4.04958100	-0.28520900	-0.59816400

O	-3.41978400	0.06216000	1.60846600

1. Bai, Y.; Kikugawa, G.; Xi, Y.; Kishimoto, N. Development of a multistep-GRRM/MC/MD simulation method for the formation of crosslinked network structures via multistep reversible reaction pathways. *Polymer* **2024**, *292*, 126606, doi:https://doi.org/10.1016/j.polymer.2023.126606.
